# Association between dental amalgam fillings and Alzheimer’s disease: a population-based cross-sectional study in Taiwan

**DOI:** 10.1186/s13195-015-0150-1

**Published:** 2015-11-12

**Authors:** Yi-Hua Sun, Oswald Ndi Nfor, Jing-Yang Huang, Yung-Po Liaw

**Affiliations:** School of Dentistry, Chung Shan Medical University, Taichung City, 40201 Taiwan; Department of Public Health and Institute of Public Health, Chung Shan Medical University, No. 110 Sec. 1 Jianguo N. Road, Taichung City, 40201 Taiwan; Department of Family and Community Medicine, Chung Shan Medical University Hospital, Taichung, Taiwan

## Abstract

**Introduction:**

The potential effects of amalgam fillings on the development of Alzheimer’s disease (AD) are not well understood. The aim of the study was to evaluate the association between dental amalgam fillings and Alzheimer’s disease in Taiwanese population aged 65 and older.

**Methods:**

Data were retrieved from the Longitudinal Health Insurance Database (LHID 2005 and 2010). The study enrolled 1,943,702 beneficiaries from the LHID database. After excluding death cases and individuals aged 65 and under, 207,587 enrollees were finally involved in the study. Dental amalgam fillings are coded as 89001C, 89002C, 89003C, 89101C, 89102C, or 89103C in the national health insurance research database (NHIRD). Alzheimer’s disease was diagnosed using the International Classification of Diseases, Ninth Revision, Clinical Modification (ICD-9-CM) codes 331.0.

**Results:**

Individuals exposed to amalgam fillings had higher risk of Alzheimer’s disease (odds ratio, OR = 1.105, 95 % confidence interval, CI = 1.025-1.190) than their non-exposed counterparts. Further analysis showed that the odds ratio of Ahlzheimer's disease was 1.07 (95 % CI = 0.962-1.196) in men and 1.132 (95 % CI = 1.022-1.254) in women.

**Conclusions:**

Women who were exposed to amalgam fillings were 1.132 times more likely to have Alzheimer’s disease than were their non-exposed counterparts.

## Introduction

Dental amalgam, a material for filling prepared cavities after removing caries, consists of about 50 % mercury [1]. Mercury vapor has been proven to be toxic to the central nervous system. In 2008, the European Commission asserted that there is no evidence showing negative effects on the human central nervous system when applying amalgam fillings as reported in previous studies. In 2009, a similar statement was made by the American Dental Association [2, 3]. The United States Food and Drug Administration, however, stated in 2008 that mercury in amalgam can increase neural risk in children and pregnant women [4]. Some scientific experiments showed that amalgam restorations in the oral cavity keep releasing human-absorbable mercury vapor [5–8]. Other studies have reported significant associations between mercury concentration in urine or in blood and quantities of amalgam restoration or number of total faces in amalgam restoration [9–11]. Furthermore, occupational studies on mercury exposure provided a strong association between mercury metal and the degeneration of the nervous system [12]. Inorganic mercury chloride (HgCl_2_) at 0.025 to 25 μM has been associated with both neuronal degeneration and perturbed excitability [13]. Hock and colleagues have reported a two-fold increase in mercury levels among patients with Alzheimer’s disease (AD) when compared to control counterparts [14]. The influence of mercury on AD is not well understood. However, it has been demonstrated that mercury can dramatically promote heparin–induced aggregation of R2, the Alzheimer’s tau fragment [15].

AD has been regarded as an unsolved, irreversible, and incurable brain neuron degeneration. AD patients normally have difficulties retrieving memory. They exhibit behavioral change as the disease progresses. Eventually, the disease leads to loss of control of body functions [16–18]. Being the most common type of dementia, AD is most often diagnosed in individuals 65-years old and older [19]. One study reported that in 2006, the worldwide prevalence of Alzheimer's disease was 22.6 million while it has been predicted that by 2050, 1 in 85 people will suffer from AD [20]. From 2000–2010, someone in America was reported with AD every 68 s. Deaths due to AD were reported at 68 % while those resulting from heart diseases were observed to have decreased [21]. According to the Taiwan Alzheimer’s Disease Association, an estimated 3.5 % of Taiwanese will suffer from dementia by 2050. AD, the most common type of dementia, is of critical importance in public health. The Alzheimer’s Association in the USA claimed there is no association between silver dental fillings (known as amalgam fillings) and AD [22]. However, the impact of amalgam fillings on the development of AD is not well understood [23, 24]. Aging is considered to be a major risk factor for AD [25, 26]. This study aimed to investigate the association between dental amalgam fillings and AD in the Taiwanese population 65-years and older.

## Methods

The study was conducted using the Longitudinal Health Insurance Databases (LHID 2005 and 2010). The databases comprise secondary data released to the public for research purposes; hence, this study was exempted from full review by the Institutional Review Board. Subjects included citizens who were ≥ 65 years at study end in 2010. The basic characteristics of the study subjects included births, gender and income. Dental amalgam fillings were coded as 89001C, 89002C, 89003C, 89101C, 89102C, and 89103C in the national health insurance research database (NHIRD), where 89001C indicates single-face amalgam restoration, 89002C indicates two-face amalgam restoration, and 89003C indicates three-face amalgam restoration. The following codes, 89101C, 89102C, and 89103C, represent single-surface, two- and three-surface amalgam restoration in specific cases. Individuals with AD were diagnosed using International Classification of Diseases, Ninth Revision, Clinical Modification (ICD-9-CM) code 331.0.

Data analyses were performed using the SAS 9.3 software package (SAS Institute Inc., Cary, NC, USA). Multiple logistic regression analysis was used to estimate the association between dental amalgam fillings and AD. The number of times and accumulated faces that a patient received amalgam fillings during 2001–2010 were regarded as independent variables while AD was considered as the dependent variable. Adjustments were made for age, gender, income, and residential region.

## Results

**Fig. 1:**
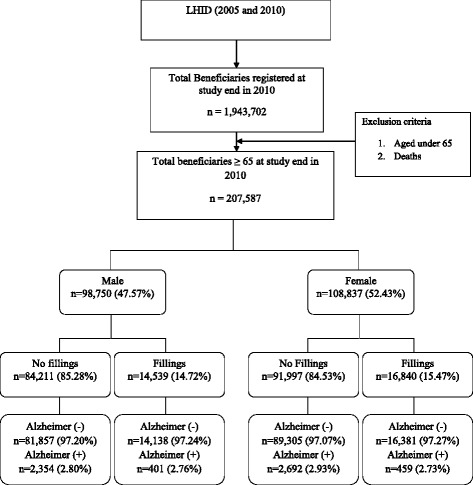
Flow chart showing amalgam fillings and Alzheimer’s disease (2000–2010)

In total, 207,587 individuals 65-years old and above were analyzed (Fig. 1). Generally, 2.76 % of men exposed to amalgam fillings and 2.8 % of their unexposed counterparts were diagnosed with AD as were 2.73 % of women exposed to amalgam fillings and 2.93 % of their unexposed counterparts. Table 1 shows the demographic characteristics of individuals with and without AD. The mean age of individuals with AD was significantly higher than of those with no AD (79.36 ± 7.14 vs. 74.87 ± 6.89, *p* < 0.001). There was no significant difference in the gender distributions of AD. Subjects with and without AD were categorized into four levels by income. The percentages of subjects with AD were 41.86, 25.70, 31.59, and 0.85 % by income ranging from 1–20,000, 20,001-40,000, and >40,000 respectively. Income distributions were significantly different among AD and non-AD individuals. Central, South and Taipei regions had high prevalences of the disease. Individuals exposed to amalgam fillings had a higher risk of Alzheimer’s disease [odds ratio (OR) = 1.105, 95 % confidence interval (CI) = 1.025-1.190] after adjusting for age, gender, income, and residential region (Table 2). Further analysis was made to assess disease risks by gender. The ORs for AD were 1.07 (95 % CI = 0.962-1.196) in men and 1.132 (95 % CI = 1.022-1.254) in women (Table 3).

**Table 1:** Baseline characteristics of study subjects

Variable	Alzheimer’s disease		No Alzheimer’s disease		*p-*value
	(*n* = 5,906)		(*n* = 201,681)		
	No.	%	No.	%	
Age (year)	79.36 ± 7.14		74.87 ± 6.89		<.0001
Sex					0.1496
Men	2,755	46.65	95,995	47.60	
Women	3,151	53.35	105,686	52.40	
Income (NT$)					<.0001
Dependent	2,472	41.86	84,740	42.02	
1–20,000	1,518	25.70	44,260	21.95	
20,001-40,000	1,866	31.59	69,890	34.65	
>40,000	50	0.85	2,791	1.38	
Region					<.0001
Area 1(Taipei)	1,659	28.09	65,573	32.51	
Area 2 (Northern)	868	14.70	26,761	13.27	
Area 3 (Central)	1256	21.27	36,693	18.19	
Area 4 (Southern)	1,247	21.11	34,690	17.20	
Area 5 (Kao-Ping)	739	12.51	32,141	15.94	
Area 6 (Eastern)	137	2.32	5,823	2.89	
Amalgam filling					0.2274
None	5,046	85.44	171,162	84.87	
Yes	860	14.56	30,519	15.13	

Age was mean ± S.E

**Table 2:** Logistic regression analysis of factors associated with Alzheimer’s disease

Variable	Logistic regression model	
	OR	95 % CI
Amalgam fillings		
None	1.000	-
Yes	1.105	1.025-1.190
Age	1.087	1.083-1.090
Sex		
Female	1.031	0.976-1.088
Male	1.000	-
Income (NT$)		
Dependent	1.374	1.287-1.466
1–20,000	1.287	1.195-1.386
20,001-40,000	1.000	-
>40,000	1.175	0.883-1.565
Region		
Area 1 (Taipei)	0.735	0.675-0.800
Area 2 (Northern)	1.000	-
Area 3 (Central)	1.127	1.032-1.232
Area 4 (Southern)	1.226	1.120-1.341
Area 5 (Kao-Ping)	0.734	0.672-0.821
Area 6 (Eastern)	0.732	0.609-0.880

Odds ratio was adjusted for age, gender, insurance level, and residential region

*OR* odds ratio, *CI* confidence interval

**Table 3:** Characteristics of people with amalgam fillings in both genders

Variable	Logistic regression model			
	Men		Women	
	OR	95 % CI	OR	95 % CI
Amalgam fillings				
No	1.00	-	1.00	-
Yes	1.07	0.962-1.196	1.132	1.022-1.254
Age	1.09	1.084-1.096	1.085	1.080-1.090
Income (NT$)				
Dependent	1.278	1.156-1.413	1.455	1.335-1.585
1–20,000	1.162	1.052-1.284	1.452	1.297 -1.625
20,001-40,000	1.00	-	1.00	-
>40,000	0.945	0.656-1.360	1.770	1.113-2.815
Region				
Area 1^a^	0.838	0.740-0.950	0.650	0.579-0.730
Area 2^b^	1.00	-	1.00	-
Area 3^c^	1.302	1.142-1.484	0.991	0.878-1.118
Area 4^d^	1.409	1.234-1.610	1.087	0.963-1.228
Area 5^e^	0.801	0.692-0.929	0.695	0.606-0.796
Area 6^f^	0.881	0.685-1.134	0.611	0.466-0.800

Odds ratio was adjusted for age, income, and residential region

^a^Area 1 (Taipei): Taipei City and County, Yilan County, Keelung City, Kinmen County, and Lienhiang County)

^b^Area 2 (Northern): Taoyuan County, Hsinchu County, Miaoli County, and Hsinchu City

^c^Area 3 (Central): Taichung City, Taichung County, Changhua County, and Nantou County

^d^Area 4 (Southern): Tainan City and County, Chiayi City and County, Yunlin County

^e^Area 5 (Kao-Ping): Kaohsiung City and County, Pingtung County, Penghu County

^f^Area 6 (Eastern): Hualin and Taitung County

## Discussion

This is the first study to investigate the association between dental amalgam fillings and AD disease using large-scale data. The study results show that women exposed to mercury amalgam fillings were 1.132 times more likely to have Alzheimer’s disease than were their non-exposed counterparts. These findings are similar to those of previous studies that have reported elevated levels of plasma mercury in subjects with AD in comparison with their healthy controls [14, 27]. Moreover, mercury has been suggested as an important toxic element in AD [28]. Since the launching of the National Health Insurance (NHI) in Taiwan, the coverage rate was 97 % by 2001 and 99 % by 2009 [29, 30]. The NHI has been responsible for the payment of amalgam fillings since 2004. The association between amalgam fillings and AD may have been underestimated considering that the number of amalgam fillings from 2001–2010 may not represent the actual fillings during the patients’ lifetimes. However, people who had multiple amalgam fillings from 2001–2010 were more likely to have subsequent fillings. The NHIRD contains dental records of every beneficiary limited to 2001 ~ 2010. Regarding the individuals without dental amalgam fillings, it is possible that this patient group may have had amalgam fillings earlier in their life, and before 2001, removed them or replaced them with other dental materials, such as gold alloys or ceramics. There are also root filled teeth in jaws. Some root fillings, especially the retrograde root fillings, often contain mercury amalgam. In this study, the control group was not a “pure” non-exposure group because its individuals may have been exposed before 2001. Of course, prior to 2001, exposure to amalgam fillings might have either been lesser or greater in the exposure than the non-exposure group, and vice versa. This also applies for replacements with other dental materials, such as gold alloys or ceramics. However, there may have been non-differential misclassification at baseline which might have resulted in underestimation. From our findings, however, the results are significant implying higher risk of exposure. Even though the NHIRD could not provide complete information on the exact number of fillings and AD, amalgam fillings from 2001–2010 could equally be representative of the total number of fillings during a patient’s life. The same assumption was made for accumulated faces. Accumulated faces are the sum of the number of faces of each amalgam filling .

AD is often diagnosed among older people. This explains why subjects 65-years old and above were used in this study. Molecular lesions have been observed in more than 80 % of senile AD brains [31]. A study showed that low income predicts the risk of developing incident AD [31]; hence, income was adjusted in this study. Environmental pollutants, such as heavy metal contaminations in ground water, contribute to varying levels of dietary mercury. Residential regions were adjusted because higher concentrations of mercury have been reported in western townships [32].

In this study, it is assumed that mercury released from amalgams would initiate AD. However, it would also be possible that oral exposure to nickel, palladium, chromium or cobalt might also be a cause of AD. Some people who visit the dentist might have a higher chance of getting amalgam restorations but also of getting cast restorations, such as crowns or bridges. In Asian countries nickel-based alloys are often used for this treatment. However, the National Health Insurance Database does not provide data on crowns and bridges partly because they are considered as advanced prosthodontic treatments for both esthetics and function. Moreover, they are more costly than amalgam restorations. The authors believe that crowns and bridges are a concern yet are less critical than amalgam restoration due to the following reasons: (1) The frequency of getting crowns and bridges is lower compared to amalgam usage. Amalga is indicated when early caries are present. Crowns are indicated when the tooth structure is severely destroyed possibly due to severe caries while bridges are indicated in edentulous areas. (2) Most of the crowns and bridges are porcelain-fused-metal. That is, they are covered by porcelain with a natural-tooth-like look. The degree of metal exposure to the oral cavity is less. Even with all-metal crowns and bridges, the surfaces of the prosthesis are highly polished to decrease the abrasion of the metals. Therefore, effects due to oral exposure to nickel, palladium, chromium or cobalt were possibly minimized.

## Conclusions

After adjusting for age, income and residential region, women exposed to mercury amalgam fillings were 1.132 times more likely to have AD than were their non-exposed counterparts.

## Abbreviations

AD: Alzheimer’s disease
CI: Confidence interval
ICD-9-CM: International Classification of Diseases, Ninth Revision, Clinical Modification
LHID: Longitudinal health insurance database
NHIRD: National health insurance research database
